# SREBP-2/PNPLA8 axis improves non-alcoholic fatty liver disease through activation of autophagy

**DOI:** 10.1038/srep35732

**Published:** 2016-10-21

**Authors:** Kwang-Youn Kim, Hyun-Jun Jang, Yong Ryul Yang, Kwang-Il Park, JeongKon Seo, Il-Woo Shin, Tae-Il Jeon, Soon-cheol Ahn, Pann-Ghill Suh, Timothy F. Osborne, Young-Kyo Seo

**Affiliations:** 1 School of Life Sciences, Ulsan National Institute of Science and Technology (UNIST), UNIST-gil 50, Ulsan, 44919, Korea; 2 Korean Medicine (KM)-Application Center, Korea Institute of Oriental Medicine (KIOM), Cheomdan-ro 70, Daegu 41062, Korea; 3 UNIST Central Research Facilities, Ulsan National Institute of Science and Technology (UNIST), UNIST-gil 50, Ulsan, 44919, Korea; 4 School of Medicine, Gyeongsang National University, Jinju, 52727, Korea; 5 Department of Animal Science, Chonnam National University, Gwangju 61186 South Korea; 6 Department of Microbiology & Immunology, Pusan National University School of Medicine, Yangsan 50612, Korea; 7 Integrative Metabolism Program, Sanford Burnham Prebys Medical Discovery Institute, Orlando, FL 32827, USA

## Abstract

Dysregulated autophagy is associated with steatosis and non-alcoholic fatty liver disease (NAFLD), however the mechanisms connecting them remain poorly understand. Here, we show that co-administration of lovastatin and ezetimibe (L/E) significantly reverses hepatic triglyceride accumulation concomitant with an increase in SREBP-2 driven autophagy in mice fed a high-fat diet (HFD). We further show that the statin mediated increase in SREBP-2 directly activates expression of patatin-like phospholipase domain-containing enzyme 8 (PNPLA8) gene, and PNPLA8 associates with autophagosomes and is associated with a decrease in cellular triglyceride. Moreover, we show that over-expression of PNPLA8 dramatically decreases hepatic steatosis through increased autophagy in hepatocytes of HFD-fed mice. Live-cell imaging analyses also reveal that PNPLA8 dynamically interacts with LC3 and we suggest that the SREBP-2/PNPLA8 axis represents a novel regulatory mechanism for lipid homeostasis. These data provide a possible mechanism for the reported beneficial effects of statins for decreasing hepatic triglyceride levels in NAFLD patients.

Obesity is becoming an increasingly important clinical and public health challenge worldwide^1^,^2^,^3^. Metabolic studies have suggested that obesity is associated with a high risk of development of life-threatening diseases such as type 2 diabetes, hypertension, coronary artery disease, and heart failure. There is also a strong relationship between lipid metabolism and many physiologic and pathophysiologic processes^4^. The mammalian liver accumulates excess lipids as a consequence of common metabolic imbalances that occur with obesity, type 2 diabetes and direct lipid disorders. This is often referred to as non-alcoholic fatty liver disease (NAFLD) and cellular lipid overload can be detrimental to normal cell function in a variety of different tissues, as described by the “lipotoxicity hypothesis”^5^. Despite general agreement that aberrant regulation of cellular lipid contributes to diverse diseases the underlying molecular mechanisms are multifaceted and remain somewhat controversial^6^,^7^. Excess lipid accumulation results from improper cellular lipid handling export, from altered synthesis, storage or oxidation in response to cellular regulatory cues. A part of the cellular adaptive response includes regulation of key lipid synthetic genes by the sterol regulatory element binding proteins (SREBPs)^8^,^9^.

SREBPs comprise a three-member transcription factor family that play key roles in lipid homeostasis^8^,^9^. SREBPs are basic helix–loop–helix leucine zipper transcription factors^10^,^11^, and mammals express three major isoforms that are encoded by two genes. The *Srebf-1* gene produces two overlapping mRNAs that differ only in their 5′-terminal exons. The resulting proteins SREBP-1a and SREBP-1c are identical, except for unique amino-termini of their transcriptional activation domains. In addition, a separate *Srebf-2* gene encodes a single SREBP-2 protein. In a previous paper, we described a genome-wide binding/ChIP-Seq analysis of SREBP-2 in mouse liver chromatin that revealed SREBP-2 occupied the promoters of several autophagy-related genes^12^,^13^. We also showed that in cholesterol-depleted cells, SREBP-2 knockdown reduced autophagosome formation and lipid droplet association with the autophagosome protein LC3. This is consistent with a more general role for SREBP-2 in autophagy to regulate lipid mobilization. However, how SREBP-2 might connect lipid breakdown with autophagy remained unclear.

Recent studies have shown that patatin-like phospholipase domain-containing enzyme 5 (PNPLA5) plays important roles in both TAG metabolism and LD homeostasis^14^,^15^. PNPLAs contain a conserved serine lipase motif (Gly-x-Ser-x-Gly) and exhibit acyl-hydrolase activity^16^,^17^,^18^. Nine PNPLA family members (PNPLA1–9) have been identified in various tissues in humans and they play important roles in various cellular processes. PNPLA3 is associated with NAFLD in humans, possesses both acyl hydrolase and synthesis activity in partially purified form, and is regulated in the liver by SREBP-1c during the insulin dependent fasting/feeding cycle^19^. PNPLA8 (also known as iPLA2γ) preferentially acts on arachidonic acid (AA) containing membrane phospholipids (PL) to generate free AA along with lysophosphatidic acid (LPA). AA can be converted into biologically active prostaglandins^20^,^21^,^22^ and there is also compelling evidence suggesting that PNPLA8 may also be involved in the regulation of signal transduction, cell growth, gene expression, and innate immune and inflammatory reactions possibly through regulation of the PI3K-TORC1 pathway^22^. Although TORC1 is a major regulator of autophagy initiation through phosphorylating ULK1/2, a direct role for PNPLA8 in autophagosome formation has not been reported.

Here, we provide evidence for PNPLA8 as a candidate enzyme that links lipid metabolism and autophagy initiation. We show that PNPLA8 is a direct SREBP-2 target gene and that induction of PNPLA8 in the livers of mice fed with a chow diet supplemented with lovastatin plus ezetimibe (L/E) or direct introduction of PNPLA8 into livers of HFD fed mice decreases hepatic lipid accumulation. We further show that PNPLA8 contributes to lipid droplet mobilization in mouse primary hepatocytes where it dynamically associates directly with autophagosomes. The SREBP-mediated induction of PNPLA8 provides a possible mechanistic understanding for the effectiveness of statin therapy in reducing hepatic lipid levels in patients with hypercholesterolemia and NAFLD^3^,^23^.

## Results

### Lovastatin attenuates hepatic lipid accumulation in a high-fat diet mouse model

**Figure 1:**
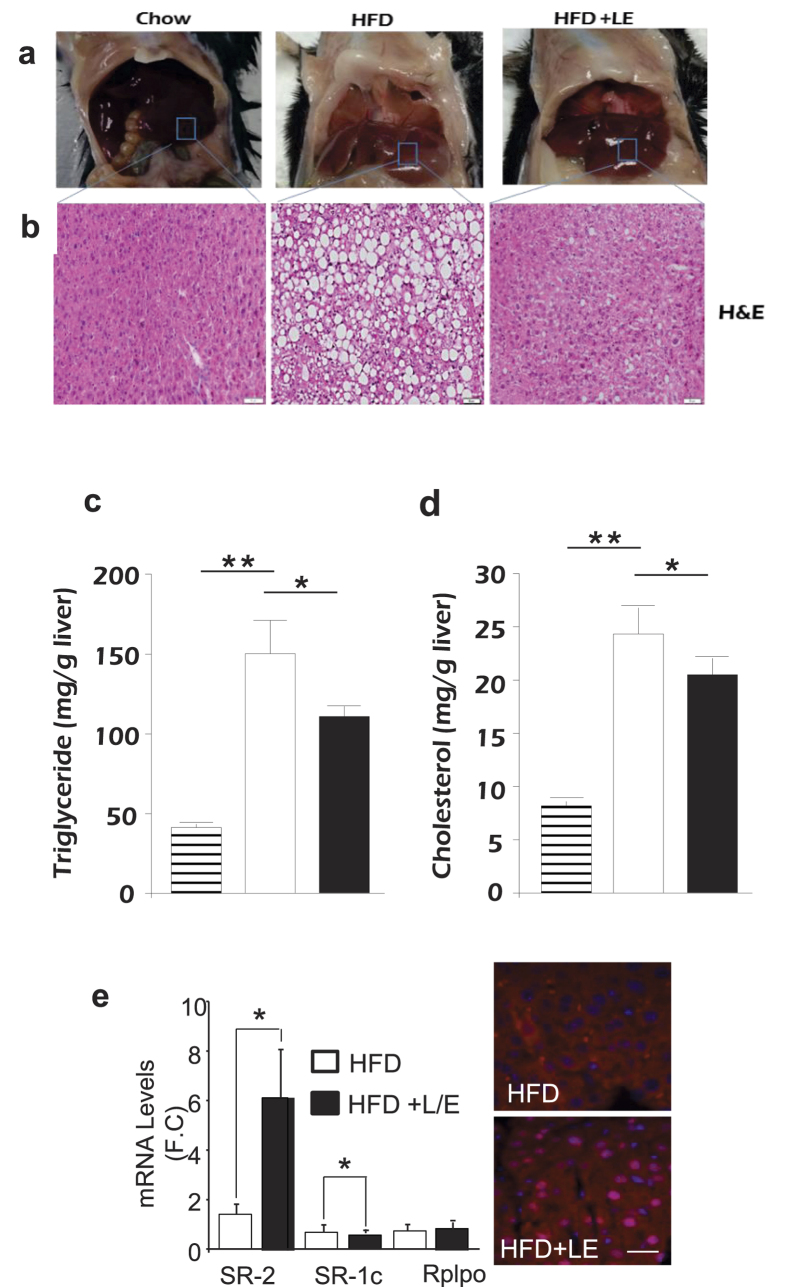
Lovastatin reverses hepatic lipid accumulation in mice fed with a high-fat diet (HFD). Mice were fed a HFD for 12 weeks followed by gavage administration of either vehicle control or a mixture of lovastatin and ezetimibe (L/E) three times (days 1, 4, 8) and all mice were sacrificed on day 12. Chow diet mice were used as basal controls. The livers from chow, HFD or HFD with L/E treated mice (**a**) and hematoxylin and eosin (H&E)-stained sections of a representative liver from chow, HFD or HFD L/E group (**b**). Hepatic triglyceride (**c**) and cholesterol levels (**d**) from the different groups are presented. Striped, open, and filled bars denote chow, HFD, and HFD + L/E groups, respectively. (**e**) SREBP-2 and SREBP-1c mRNA levels were analyzed in mice fed with HFD (open bars) or HFD + L/E (filled bars). Immunohistochemical analysis of livers from mice fed with HFD or HFD plus L/E using an anti-SREBP-2 antibody was performed as described in materials and methods. All data are representative of at least three independent experiments. Student’s t-test was used for the statistical analysis. Data are shown as means ± standard deviations. *p < 0.05, **p < 0.01.

In a prior study, we showed that treating chow fed mice with lovastatin plus ezetimibe (LE) increased expression of autophagy genes through SREBP-2^12^. To determine how this might alter hepatic lipid storage, we examined the effects of LE treatment on hepatic lipid accumulation in mice consuming a high- fat diet (HFD). Therefore, mice were fed a HFD for 12 weeks and separated into two groups and the HFD was continued except one group was supplemented with an oral gavage of L/E to inhibit cholesterol production in the liver and simultaneously limit cholesterol uptake from the diet. The other group was treated with a vehicle as a control. Mice were gavaged every third day and sacrificed after a total of 12 days. Gross evaluation showed the pale liver appearance consistent with excess hepatic lipid accumulation in the control mice (Fig. 1a). Hematoxylin-eosin (H&E) staining and direct measurements of hepatic lipid levels were consistent with reduced lipid overload in the LE treated group (Fig. 1b–d). As expected, SREBP-2, but not SREBP-1c, mRNA and nuclear protein levels were increased by the LE treatment as well (Fig. 1e). Taken together, these results suggest that SREBP-2 induction is associated with a decrease in hepatic lipid in response to the nutrient overload of HFD.

Because our prior study suggested SREBP-2 might increase lipid droplet turnover by activating genes of autophagy and depletion of lipids has been shown to increase autophagy, we proposed that SREBP-2 activation in response to LE supplementation might induce autophagy to limit HFD associated liver lipid accumulation.

### Statin induces autophagy in primary hepatocytes

**Figure 2:**
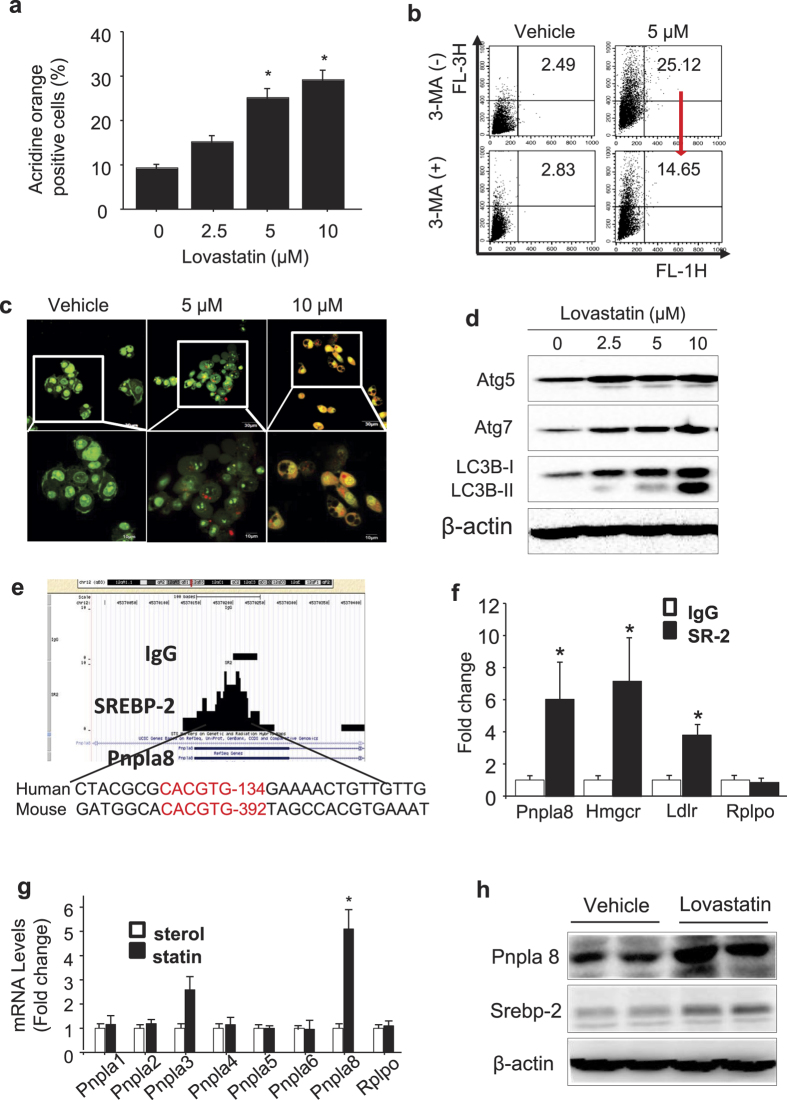
Statin induces autophagy in primary hepatocytes and Pnpla8 is upregulated by SREBP-2. Mouse primary hepatocytes (MPH) were prepared from HFD treated mice and dishes were incubated with different concentrations of lovastatin for 24 hr and then analyzed. (**a**) Acidic vacuoles (AVOs) were measured using acridine orange (AO) staining. Cells were analyzed by flow cytometry and the percentage of AO positive cells was determined. (**b**) Dishes were treated with vehicle or 1 mM 3-MA for 1 h prior to AO staining and flow cytometric evaluation. The red arrow indicates AO staining was reduced from 25.12% to 14.65% by 3-MA. (**c**) Dishes were prepared for confocal imaging of AO staining acidic vacuoles (red) and live cells (green). (**d**) Cell extracts were analyzed by immunoblotting to measure the autophagy-related proteins Atg5, Atg7, and LC-3B. (**e**) A genome browser view of SREBP-2 ChIP-Seq read mapping data (Seo *et al.*^12^) (11) detected a peak around SREBP-2 consensus sequence within the *Pnpla8* promoter (chr12:45370150-45370250). (**f**) Gene-specific manual ChIP analysis in hepatic chromatin from HFD + L/E-treated group was performed using an antibody against SREBP-2 (filled bars) or control IgG (open bars). Gene-specific primers for known SREBP binding peaks in the indicated promoter regions were used in the qPCR. Data are shown as means ± standard deviations. *p < 0.05. All data are representative of three independent experiments. (**g**) RNA harvested from mouse primary hepatocytes from HFD treated mice cultured for 24hr in the presence of sterols (open bars) or the absence of sterols plus 5 uM lovastatin for 24 hr. (filled bars) was subjected to *Pnpla* gene expression profiling via q-PCR. Target gene expression levels were normalized to levels of the housekeeping gene *Rplpo* using the ΔCt method. (**h**) MPH from HFD treated mice were cultured for 24 hr with media containing sterols or sterol depleted media containing 5 uM lovastatin and PNPLA8 protein were measured.

To evaluate the effects of statins on autophagy and hepatic lipid accumulation, we treated primary hepatocytes from HFD fed mice with increasing concentrations of lovastatin and measured autophagy using acridine orange (AO) which turns from green in healthy cells to orange as it accumulates in acidic vesicular organelles (AVOs). Figure 2a shows that the percentage of AO-positive primary hepatocytes detected via flow cytometry, was increased by lovastatin in a dose-dependent fashion. When MPH were treated with the autophagy inhibitor, 3-methyladenine (3-MA), the percentage of statin induced AO positive cells declined from 25.12% to 14.65% (Fig. 2b). Next, AVO formation was also analyzed in cells via confocal microscopy. Vehicle-treated cells exhibited green fluorescence with minimal red fluorescence, whereas cells treated with 5 or 10 μM statin exhibited an increase in red fluorescence consistent with elevated AVO formation (Fig. 2c). In addition, the autophagy-specific markers Atg5, Atg7, and LC3B were also increased as measured via immunoblotting analysis (Fig. 2d). Taken together, these results suggest that statin treatment of mouse hepatocytes increases autophagy. Because LC3 accumulation can also occur when autophagy is blocked, we evaluated the effects of PNPLA8 and statins on triglyceride levels in HepG2 cells after treatment with palmitic acid to induce autophagy. We transfected cells with aiRNAs to knockdown PNPLA8 prior to palmitic acid treatment. After 24 hr of palmitic acid (400 uM, treatment), HepG2 cells were treated with statins (5 uM) in the presence and absence of chloroquine (50 uM) and endogenous triglyceride (TG) levels were measured over the time points of 0 to 16 hr. Consistent with lipid turnover through autophagy, chloroquine treatment blocked the decrease in TG levels observed similar to what occurred when PNPLA8 was reduced through si-RNA mediated knockdown (Supplementary Fig. S1a). We also measured LC3B conversion in this experiment and consistent with statin treatment leading to increased autophagic flux, LC3IIB levels were further increased by chloroquine (Supplementary Fig. S1b).

### SREBP-2 regulates expression of PNPLA8

We previously reported the genome-wide binding patterns of SREBP-2 in hepatic chromatin and uncovered a preferred binding motif^12^,^24^. To find a SREBP-2-regulated gene that might contribute to lipid hydrolysis and be responsible for the decrease in hepatic lipid accumulation following LE treatment in HFD mouse liver, we reviewed our previous genome-wide analysis of SREBP-2 localization and found a highly enriched SREBP-2 binding peak at the *Pnpla8* promoter (Fig. 2e). A gene-specific ChIP analysis with chromatin from statin treated primary hepatocytes and primers flanking this region confirmed SREBP-2 binding to the Pnpla8 gene promoter (Fig. 2f). The binding enrichment was comparable to SREBP-2 binding at known SREBP-2 regulated the promoters for HMGCR and LDLR (Fig. 2f). To determine whether SREBP-2 activates expression of PNPLA8 or any other PNPLA family members, we measured expression of 7 PNPLA-encoding genes in control and statin treated primary hepatocytes and as shown in Fig. 2g, *Pnpla8* and *Pnpla3* mRNA expression levels were both induced by statin treatment. PNPLA8 protein levels were also increased by statin treatment (Fig. 2h). We previously reported that SREBP-1 binds and activates the PNPLA3 promoter, however, this is the first demonstration that SREBPs might activate expression of PNPLA8. The sequence comparison in Fig. 2e suggests the human PNPLA8 gene might also be SREBP responsive, thus we transfected a FLAG epitope-tagged SREBP expression vector into human 293T cells and measured the endogenous levels of PNPLA8 proteins via Western blot analysis. We found that the accumulation of nuclear FLAG-tagged SREBP-2 was accompanied by an increase in both PNPLA8 protein and mRNA levels (Supplementary Fig. S2a,b).

### PNPLA8 induction reduces lipid accumulation in hepatocytes from HFD liver

**Figure 3:**
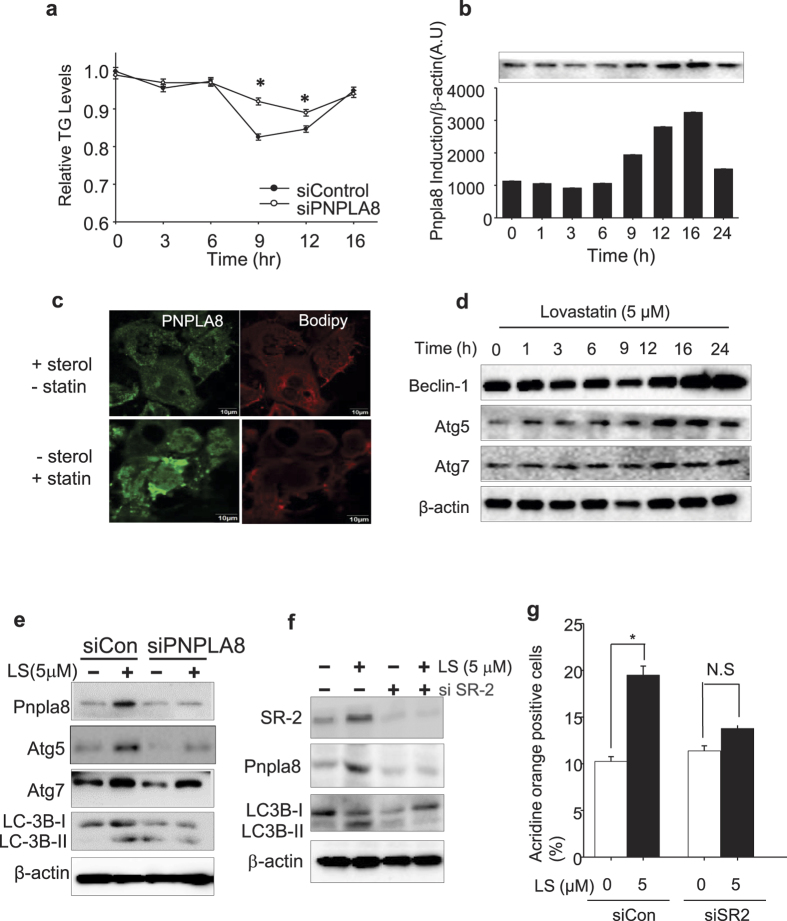
PNPLA8 expression correlates with lipid droplet depletion in primary hepatocytes. MPH from HFD treated mice were transfected with a control or PNPLA8 siRNA for 24 hr and then cultured with medium supplemented with lipid-depleted media and lovastatin (final 5 uM) for the indicated times. (**a**) Triglyceride levels (TG) were measured in total cell lysates and plotted as a relative level of the starting value which was 154 mg/g protein. (**b**) Pnpla8 protein levels were measured as indicated time points. (**c**) The MPH were cultured with ( + ) or without (−) statin as shown in a and evaluated for the localization of lipid droplets (BODIPY 493/503, red and PNPLA8 (green). (**d**) Whole cell lysates were analyzed for autophagy related-proteins via immunoblotting. (**e**,**f**) Immunoblots for autophagy-related proteins from primary hepatocytes cultured as above and treated for 24 hr with si-control or si-PNPLA8. (**g**) The AO positive cells with (5 uM) or without lovastatin were compared to siSREBP2 –treated dish. All data are representative of three independent experiments. Data are shown as means ± standard deviations. *p < 0.05.

Next, we analyzed how activation of endogenous SREBP-2 in primary hepatocytes would influence the endogenous expression of PNPLA8, lipid accumulation, and autophagy. Primary hepatocytes from HDF mice were transfected with a control siRNA or a siRNA targeting PNPLA8 and then cultured in medium without sterols but with lovastatin (5 μM) and endogenous triglyceride levels were followed over the course of 16hr. (Fig. 3a). The statin treatment resulted in a transient increase in PNPLA8 protein (Fig. 3b) and this was accompanied by a transient decrease in cell TG levels (Fig. 3a). Importantly, the knockdown of PNPLA8 prevented the decrease in TG similar to what we observed previously when SREBP-2 was depleted in a similar experiment^12^. When the effect of statin treatment on PNPLA8 expression was monitored using immunofluorescence and cell lipids were analyzed via the fluorescent lipophilic dye BODIPY, we observed that an increase in PNPLA8 expression was associated with a reduction in bodipy staining lipid droplets (Fig. 3c). Figure 3d confirms that autophagy markers were increased by statin treatment and a knockdown of PNPLA8 blunts the induction (Fig. 3e). An siRNA knockdown of SREBP-2 blunted the induction of PNPLA8 by statin treatment and also reduced the number of acridine orange positive cells (Fig. 3f,g). This is consistent with SREBP-2 acting upstream of PNPLA8 in the statin dependent induction of autophagy.

### PNPLA8 regulates lipid metabolism *in vivo*

**Figure 4:**
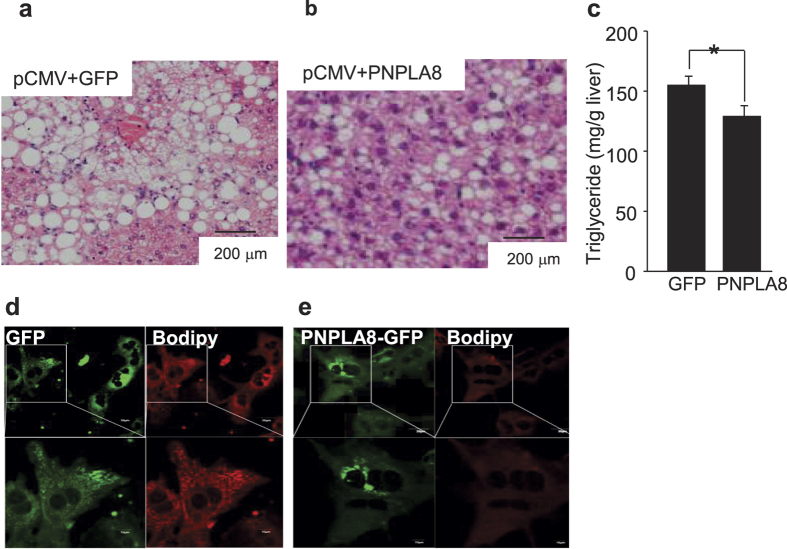
Tracking of the functional properties of Pnpla8 *in vivo.* Mice were sacrificed 12 days after transfection with control GFP (**a**,**d**) or PNPLA8-GFP expressing plasmids (**b**,**e**). Hematoxylin and eosin (H&E)-staining (**a**,**b**) or con-focal GFP and Bodipy imaging (**d**,**e**) were performed. Triglyceride (TG) were measured from the tissues lysates (**c**). Student’s t-tests were used for the statistical analysis. *p < 0.05.

To determine whether altered expression of PNPLA8 modulates lipid accumulation and autophagy in mouse liver, we injected vectors expressing GFP as a control or a PNPLA8 – GFP chimeric protein into mice using *in vivo*-jetPEI (see Materials and Methods). Briefly, mice were fed HFD for twelve weeks and then 100 μg plasmid DNA/100 μl was delivered via tail vein injections on days 1, 4 and 8 and mice were sacrificed on day 12. Hepatocytes were isolated and expression of PNPLA8-GFP in the hepatocytes was confirmed by fluorescent microscopy (Supplementary Fig. S3). Histological analysis (H&E staining) and biochemical measurements were consistent with PNPLA8 expression leading to a decrease in hepatocyte TG (Fig. 4a–c).

To visualize the intracellular distribution of lipid droplets more directly, confocal live cell imaging was performed in MPH isolated from control and GFP-PNPLA8 injected HFD fed mice using BODIPY to visualize lipid droplets and GFP fluorescence to localize PNPLA8. The BODIPY fluorescence levels were much lower in images of PNPLA8-overexpressing hepatocytes than in images of control hepatocytes (Fig. 4d,e) consistent with PNPLA8 expression leading to a reduction in intracellular lipid TG levels.

### PNPLA8 induces hepatocyte autophagy

**Figure 5:**
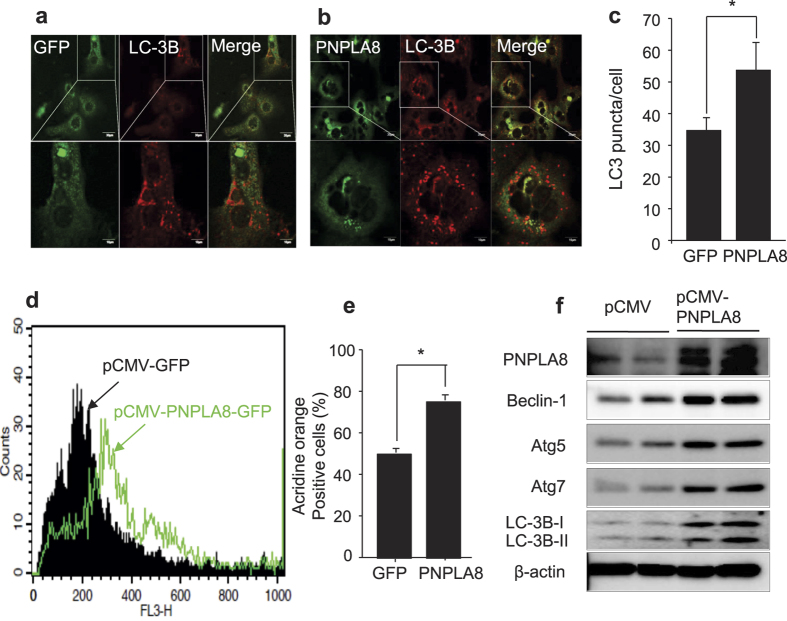
PNPLA8 induces autophagy in mice fed with high-fat diet. The control GFP (**a**) or PNPLA8-GFP (**b**) expression vectors were injected into mice after 12 weeks of HFD fed and the MPH were prepared and analyzed for GFP and LC3B staining. Confocal imaging for GFP (green) and LC3B (red). (**c**) The number of LC3B puncta per cell were calculated from 10 cells, *p < 0.05. (**d,e**) Flow cytometric detection of acidic vacuoles (AVOs) of the MPH from a. (**f**) Immunoblots for autophagy-related proteins. Each lane is from a separate dish of cells from each condition analyzed separately. Data are shown as means ± standard deviations. *p < 0.05.

To investigate whether over-expression of PNPLA8 directly affects autophagy, the formation of LC3 puncta was compared in primary hepatocytes from GFP or PNPLA8-GFP expressing livers using confocal imaging for GFP and LC3B. The results in Fig. 5a–c show a 40% increase in LC3B puncta in PNPLA8 expressing sample. Next, autophagy was analyzed by accumulation of AVOs by flow cytometry following AO staining (Fig. 5d,e). There was a 30% increase in AO positive cells in the PNPLA8-expressing cells (Fig. 5e). Consistent with an increase in autophagy, expression of autophagy markers Beclin-1, Atg5, Atg7, and LC3B were all elevated in PNPLA8 expressing hepatocytes as demonstrated via immunoblotting analysis (Fig. 5f).

### The serine lipase motif of PNPLA8 is required for lipid mobilization but not autophagosome association

**Figure 6:**
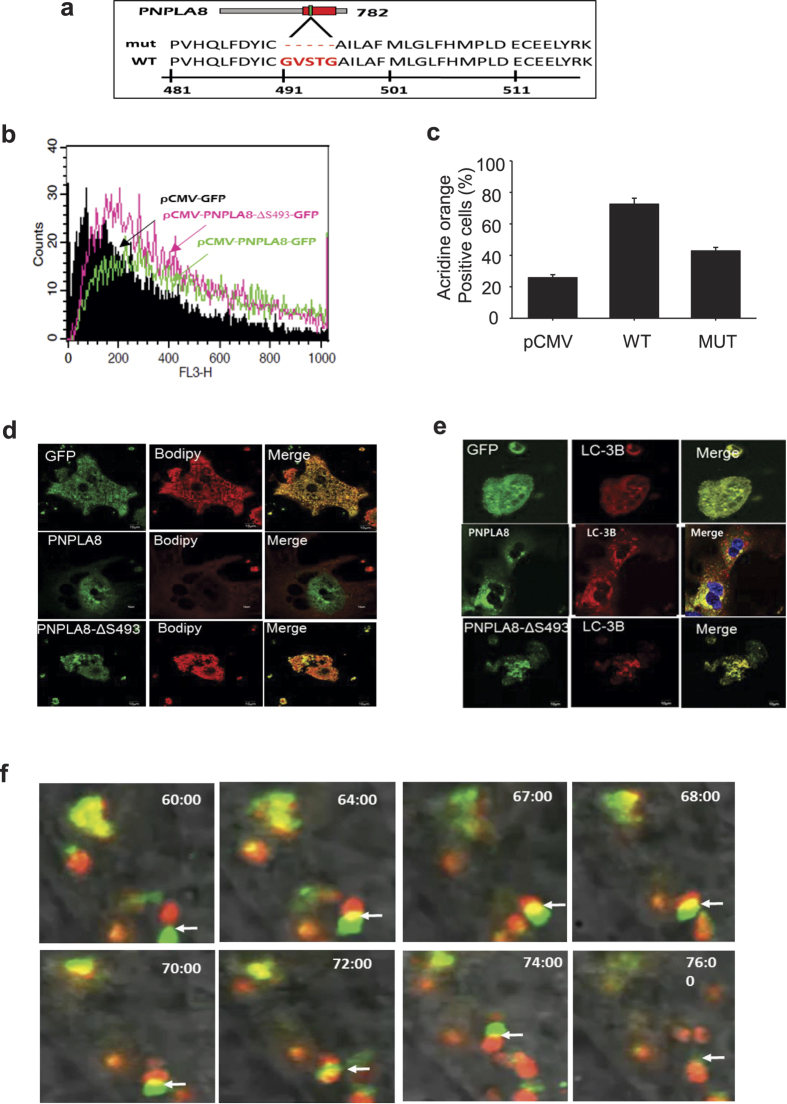
PNPLA8 consumes lipid droplets and induces autophagy. (**a**) Deletion cartoon (upper panel) of serine lipase motif (Gly-x-Ser-x-Gly) from PNPLA8 amino acid sequence (lower panel). (**b,c**) Primary hepatocytes from mice fed with high-fat diet were cultured and acidic vacuoles (AVOs) from GFP vector, PNPLA8 + GFP (WT) or PNPLA8 ΔS493 (Mut) transfected primary hepatocytes were analyzed by FACS. (**d**) Confocal images of PNPLA8-GFP (green) and Bodipy (red) from pCMV + GFP, WT-, and Mut-transfected primary hepatocytes were shown, respectively. (**e**) Confocal images of PNPLA8-GFP (green) and LC3B (red) from GFP, WT-, and Mut-transfected primary hepatocytes were shown. (**f**) Time laps images show dynamic interactions between PNPLA8 and LC3 indicating an interaction between a GFP-PNPLA8-positive structure (green) and LC3B (red) in a lipid rich cell line. Images were acquired at the times indicated. Images are representative from 3 separate experiments.

Pnpla8 contains a conserved serine lipase motif (Gly-x-Ser-x-Gly), which is required for its acyl hydrolase activity^18^. To explore whether the lipase motif of Pnpla8 was required for lipid droplet association and autophagy induction, we deleted the serine lipase motif in an otherwise WT cDNA (pCMV-PNPLA8-ΔS493-GFP) (Fig. 6a) and evaluated the effects on AVO formation using AO. Figure 6b–d show the lipase motif is required for AVO formation and a reduction in cellular bodipy staining. However, interestingly, the deletion of the lipase motif did not impair co-localization with LC3B (Fig. 6e) indicating that PNPLA8 can associate with autophagosomes independently of inducing lipid turnover.

To evaluate the PNPLA8-LC3B interaction dynamically in living cells, we performed time-lapse video microscopy with cells co-expressing labeled GFP-PNPLA8 (green) and RFP-LC3 (red). Still images captured over time (Fig. 6f) from the videos available in Supplementary Video S1a and b show that PNPLA8 and LC3 interact transiently in a highly dynamic fashion.

**Figure 7:**
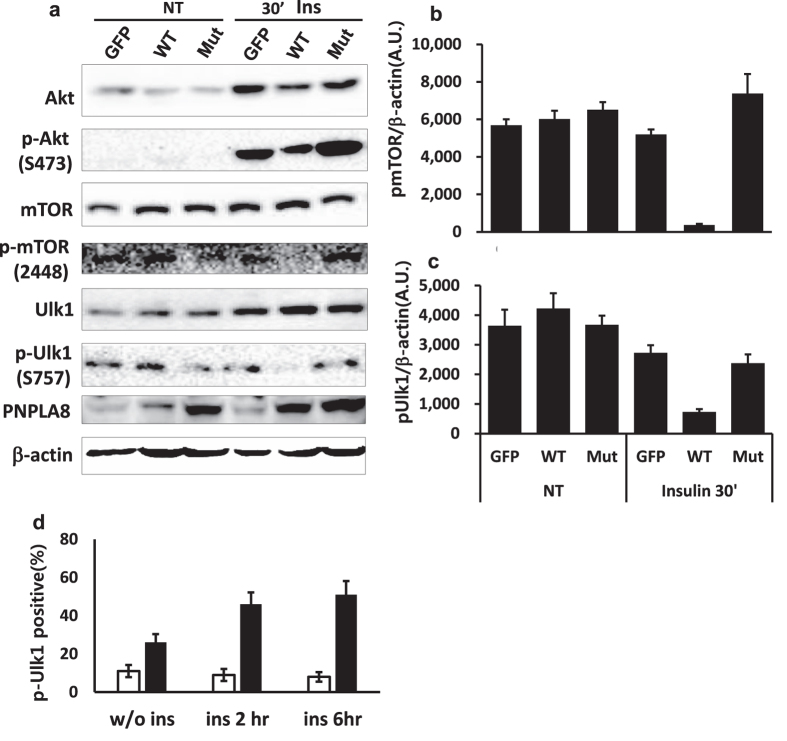
PNPLA8-mediated autophagy was regulated by mTOR inhibition. (**a**) 293T cells were transfected by the indicated plasmids. Following transfection, cells were cultured in medium with (10 μg/ml) or without insulin and harvested in 30 min. The cell lysates were prepared and total and phosphorylated forms of the indicated proteins were analyzed by western blot. β-actin was used as a sample loading control. (**b,c**) The densitometries for pmTOR and pUlk1 blots from a were compared as β-actin blot as a control (**d**) Quantitative analysis of p-Ulk1 positive stains in HEK293 cells expressing GFP as a control, WT, or Mut. Cells were fixed and stained with anti-pUlk1 (S757) antibody before being analyzed by confocal microscopy. Each bar indicating the percent of p-Ulk1 positive stains in WT expressing cells (open bars) or in Mut expressing cells (filled bars) is relative to p-Ulk1 positive of GFP expressing cells. Percentage of p-Ulk1 positive cells were calculated as follow: (p-Ulk1 in WT cells/pUlk1 in control cells) × 100 or (p-Ulk1 in Mut cells/pUlk1 in control cells) × 100.

Pnpla8 preferentially releases arachidonic acid and LPA from PL hydrolysis^18^. These are signaling lipids that are known to regulate PI3K signaling. Because PI3K signaling through TORC1 leads to phosphorylation and inactivation of the autophagy initiator protein ULK1, we reasoned that PNPLA8 might activate autophagy through inhibiting ULK1 phosphorylation downstream of TORC1. Consistent with this model, the phosphorylation of AKT, mTOR (Fig. 7a,b) and ULK1 (Fig. 7a,c) in insulin treated cells was significantly reduced by co-transfection of wild type PNPLA8 but not the mutant form lacking the lipase motif. This was also confirmed by comparing p-ULK1 GFP localization in cells transfected with the GFP tagged WT PNPLA8 versus the lipase deficient mutant (Fig. 7d). Taken together, these results suggest PNPLA8 inhibits the activation of mTOR, which would lead to reduced phosphorylation of ULK1, and increased autophagy.

## Discussion

The connection between lipid depletion and autophagy has been known for a decade and the induction of autophagy directly by statin treatment has also been widely reported^23^,^25^,^26^. However, the underlying molecular mechanism is not well understood. In a previous report, we analyzed the binding of SREBP-2 to hepatic chromatin prepared from livers of mice fed a chow diet supplemented with lovastatin and ezetimibe (LE)^12^. Interestingly, we demonstrated that SREBP-2 binds to and activates expression of several autophagy genes. Because SREBP-2 activity is increased upon low sterol levels to stimulate expression of genes required to provide the cell with new lipid, we reasoned that SREBP-2 activation of genes that promote lipid droplet mobilization through autophagy would provide the cell with new lipid quickly and without the need for the energy and time consuming processes of uptake and de novo synthesis.

Based on this model we hypothesized that adding LE to mice fed a high fat diet might reverse hepatic steatosis through increased autophagy mediated by SREBP-2 activation. Here, we provide evidence that LE induced SREBP-2 activates autophagy leading to reduced lipid accumulation in the mouse liver. The results in Fig. 1 show that hepatic triglyceride levels are significantly reduced when HFD fed mice are supplemented with LE and this is accompanied by an increase in SREBP-2. Consistent with this idea, a recent report showed that LE supplementation reversed the cholesterol accumulation and liver NASH like symptoms in a mouse model fed an atherogenic diet^27^.

Our studies also show that PNPLA8, a member of the patatin family of phospholipases, is an SREBP-2 target gene and plays a pivotal role in LD turnover in response to LE induced SREBP-2. Furthermore, when PNPLA8 was over-expressed in the liver of HFD fed mice, hepatic triglyceride levels were significantly lowered suggesting PNPLA8 was sufficient to initiate autophagic mobilization of hepatic stored triglycerides. We show that PNPLA8 interacts directly with autophagosomes and a mutation that removed its lipase motif reduced lipid mobilization but did not affect autophagosome targeting. Using time-lapsed imaging we also show evidence for a dynamic interaction between PNPLA8 and LC3B during autophagy induction.

Other members of the PNPLA family have been shown to contain LD targeting motifs and to be involved in LD mobilization^28^,^29^. PNPLA2, also called ATGL, associates with LDs and is the rate determining enzyme in triglyceride lipolysis^30^. PNPLA5 has been proposed to mobilize LD lipids for formation of the autophagosmal membrane^13^ and a PNPLA3-148M variant associates with lipid droplets and results in triglyceride accumulation associated with NAFLD^31^. However, this is the first report to demonstrate a role of PNPLA8 in LD turnover through activation of autophagy. PNPLA8 was first identified as a mitochondrial associated phospholipase but it has also been associated with the endoplasmic reticulum^32^ and now through our studies with the autophagosome. The phospholipase activity of PNPLA8 produces Lysophosphatidic acid from glycerophospholipids and also releases a fatty acyl chain from the sn-2 position. PNPLA8 preferentially releases polyunsaturated fatty acids including arachidonic acid^33^, which can be converted into a variety of intracellular signaling lipids in addition to being hydrolyzed to free fatty acids for remodeling as well as oxidation. PNPLA5 has been proposed to release diacylglycerol from LD triglyceride that is converted into phospholipids through choline phosphotransferase 1 to provide PL for formation of the autophagosomal membrane. This would spare organellar and plasma membranes from lipid loss during autophagy. While the exact role played by PNPLA8 in lipid droplet mobilization through autophagy needs further investigation, it is possible that the lipid reaction products released through sn-2 hydrolysis generates a signaling lipid that alters mTORC1 ability to inactivate autophagy through phosphorylation and inactivation of the autophagy initiator kinase ULK1. Consistent with this model, we showed that PNPLA8 over-expression resulted in an inhibition of ULK-1 phosphorylation, which would lead to an increase in autophagy. This is also consistent with the recently reported increase in p-ULK1 observed in statin treated cardiomyocytes^34^. Thus, PNPLA8 may regulate autophagy initiation as well as LD turn over through inhibition of TORC1. It is also possible that the lysophosphatidic acid generated by PNPLA8 serves as a substrate for LPCAT dependent phospholipid synthesis which would augment autophagosomal membrane biogenesis. PNPLA8 is also subject to negative feedback regulation by long chain fatty acids^33^, a property that would make sense for an enzyme involved in activating LD turnover in response to lipid depletion.

Taken together, our study suggests that PNPLA8 plays an active role in lipid turnover through the autophagosome. Because PNPLA8 is activated by SREBP-2, our results also provide a mechanism for he beneficial pharmacologic targeting of SREBP-2 by statins in patients with NAFLD.

## Materials and Methods

### Animal Care

All animal experiments were performed in accordance with accepted standards of animal welfare and with approval by Institutional Animal Care and Use Committee of the Ulsan National Institute of Science and Technology IACUC, Ulsan, Korea (protocol UNISTIACUC-14-032). C57BL/6 mice were maintained on a standard rodent chow diet with a 12-h light/dark cycle. Mice were separated into two groups of six animals per group. All mice were fed with HFD or with a normal diet (Research Diets, New Brunswick, NJ, USA). On a caloric basis, the HFD (product #; D12492) contained 60% fat from lard, 20% carbohydrates, and 20% proteins (total: 23.4 kJ/g), whereas the normal diet(product #; D12450B) contained 10% fat, 70% carbohydrate, and 20% protein (total: 12.6 kJ/g). After 12 weeks, one group of HFD mice continued on the HFD; the other two groups were fed either oral gavage administration with L/E [100 mg lovastatin (2.5 tablet equivalents) and 21 mg ezetimibe (2.1 tablet equivalents)/100 g chow, w/w]. All mice were sacrificed by CO_2_ asphyxiation at 8 am (at the end of the dark cycle). For plasmid delivery *in vivo*, the indicated plasmids (100 μg with *in vivo*-jetPEI DNA Delivery Reagent (PolyPlus, New York, NY, USA) were injected into the tail veins of mice after a 12-week HFD feeding, according to the manufacturer’s instructions.

### Triglyceride and total cholesterol analysis

Liver tissues were homogenized in buffer containing protease inhibitors and centrifuged at 10,000 × g for 10 min at 4 °C. The supernatants were transferred to fresh tubes. Blood samples were allowed to clot for 30 min at 25 °C and then centrifuged at 2,000 × g for 15 min at 4 °C. The top yellow serum layer was removed via pipette without disturbing the white buffy layer. TG and total cholesterol levels were measured in the soluble extracts from tissue or blood using a colorimetric assay kits (Cayman Chemical Company, Ann Arbor, MI, USA) according to the manufacturer’s instructions.

### Hepatocyte preparation and culture

Male C57BL/6 mice fed with a 10-week HFD were used for primary hepatocyte isolation in all experiments. Primary mouse hepatocytes (MPH) were harvested using two-step collagenase perfusion^35^. Briefly, each animal was anesthetized with avertin (250 mg/kg) and rompun (10 mg/kg). The liver was perfused by sequentially injecting pre-warmed 2.5 mM EGTA (pH 7.4), a digestion buffer (0.5 mg/mL), collagenase type IV (Sigma, St. Louis, MO, USA), 66.7 mM NaCl, 6.7 mM KCl, 50 mM HEPES (pH 7.6), and 4.8 mM CaCl_2_ into the portal vein over a 15-min period. The digested liver was removed and shaken in 199/EBSS medium (Thermo Scientific, Barrington IL, USA) supplemented with 10% fetal bovine serum (Gibco, Grand Island, NY, USA), 1 U/mL penicillin/streptomycin (Gibco), and 10 nM dexamethasone. The resulting cell suspension was filtered through a 70-μm cell strainer (BD Biosciences, San Diego, CA, USA), and the cells were pelleted and resuspended in 5 ml of fresh hepatocyte culture medium. The cells were overlaid on a 3% percoll solution (pH 7.4) and centrifuged at 700 rpm. The pelleted cells were resuspended in hepatocyte culture medium. MPH with a viability exceeding 95% (trypan blue exclusion analysis) were cultured further. The cells were seeded at a density of 4 × 10^5^ cells/well of a 6-well plate and maintained in a humidified incubator at 37 °C with an atmosphere containing 5% CO_2_ and treated as described in the figure legends.

### RNA isolation and real-time quantitative PCR

Total RNA was isolated from mouse livers and PC-3 cells using TRIzol (Invitrogen, Carlsbad, CA, USA) and QIAGEN RNAeasy isolation kits (QIAGEN, Valencia, CA, USA). Quantitative PCR reactions were performed using SYBR green fluorescent dye and a Roche 480 (Roche, Basel, Switzerland). Relative mRNA expression levels were determined using the ΔΔ-Ct method and normalized to the levels of TATA-binding protein (TBP) mRNA. The sequences of all primers used in this study are listed in Supplementary Table S1.

### Chromatin preparation for chromatin immunoprecipitation (ChIP) assays

Mouse liver chromatin preparations for ChIP assays were performed as previously described^24^,^36^. For gene-specific ChIP, qPCR to determine SREBP-2 binding to specific gene promoters was analyzed in triplicate using a standard dilution curve of the input DNA performed in parallel, and enrichment was measured by SYBR green incorporation on a Roche 480. Analysis was performed using the standard curve method, and values were normalized to a non-target control region from the ribosomal *Rplpo* gene.

### Cell culture and RNA interference

MPH were maintained in Dulbecco’s modified Eagle’s medium (DMEM) supplemented with 10% heat-inactivated fetal calf serum and antibiotics at 37 °C in an atmosphere with 5% CO_2_. PNPLA8 specific- siRNA (SR309408; Origene, USA) and control siRNAs (sc-37007) were purchased from Santa Cruz Biotechnology (Santa Cruz, CA, USA). Cells were transfected for 24 h with 10 nM of each siRNA and Lipofectamine RNAiMAX reagent (Invitrogen) according to the manufacturer’s instructions; subsequently, plates and chamber slides were washed and refilled with fresh media with or without serum for 24 h followed by analysis.

### Immunoblotting

A polyclonal rabbit antibodies were carefully evaluated their selectivity and specificity for SREBP-2 or SREBP-1 in our previous papers^12^,^24^,^36^. Hepatic nuclear protein and whole-cell lysates were subjected to sodium dodecyl sulfate-polyacrylamide gel electrophoresis (7%, 10%, or 12%), followed by transfer to nitrocellulose membranes and blocked with Tris-buffered saline with 0.02% Tween (TBST) containing 5% milk for 2 h. Membranes were incubated overnight with primary antibodies against AKT (Cell Signaling, 9272), phospho-AKT (S473; Cell Signaling, #9271), Ulk1(Cell signaling, #8054S), phospho-Ulk1 (Ser757; Cell Signaling, #6888S), and mTOR (Santa Cruz Biotechnology, sc-8319), phospho-mTOR (S2448; Santa Cruz Biotechnology, sc-101738). Membranes were washed in TBST and incubated with appropriate secondary horseradish peroxidase-conjugated antibodies (Bio-Rad, Richmond, CA). All images were acquired from Image Lab (Ver. 4.1) of ChemiDoc Imaging System (BIO-RAD, USA) and transformed in AutoScale where Gamma ray was 1.0. Each WB image as shown as representative figure was actually cropped and displayed in the main figures.

### PNPLA8-GFP and mutant-GFP plasmid constructs for detecting Ulk1 activity

cDNAs encoding PNPLA8 or GXSXG-deleted PNPLA8 were cloned into pCMV-GFP vector according to the manufacturer’s instruction. HEK-293 cells were transfected with the DNA constructs (control pCMV-GFP, pCMV-WT-GFP, and pCMV-Mut-GFP) after seeding in 6-well plates following the manufacturer’s protocol. After 24 hr post-transfection, the cells were treated with insulin for 30 min to final concentration of 5 ug/ml. For detection of Ulk phosphorylation, cells were and washed twice with ice-cold PBS. Subsequently, cells were lysed in buffer consisting of 50 mM Tris-HCl, pH 8.0, 5 mM EDTA, 150 mM NaCl, 0.5% sodium deoxycholate, 1% Nonidet P-40 (Sigma-Aldrich, 74385), 0.1% sodium dodecyl sulfate, 1 mM PMSF, 1 mM sodium fluoride, 1 mM sodium orthovanadate, and protease inhibitor cocktail. For ULK1 localization, HEK293 cells harboring the plasmid constructs were fixed and imaged for GFP fluorescence as followed.

### Immunofluorescence and immunohistochemistry

Sections of paraffin-embedded livers were dewaxed in xylene, rehydrated, and subjected to antigen retrieval in 10 mM citrate buffer. Sections were blocked for 30 min in 1% bovine serum albumin, 0.02% Triton X-100, and 10% normal goat serum (NGS). Serial sections were incubated with rabbit polyclonal SREBP-2 antibody (1:500 dilution), followed by Cy5-conjugated goat anti-rabbit secondary antibody (Jackson ImmunoResearch Laboratories, Inc., West Grove, PA, USA). The sections were mounted using Vectashield with DAPI (Vector Laboratories, Burlingame, CA, USA). Images from serial sections were acquired using an Axioskop inverted microscope with an AxioVision camera and software (Zeiss, Thornwood, NY, USA). For immunocytochemistry, PC-3 cells were fixed with 4% paraformaldehyde, permeabilized with 0.25% Triton X-100, blocked with 10% NGS, and incubated with anti-LC3B primary antibody, followed by Alexa Fluor 488-conjugated goat anti-rabbit secondary antibody before mounting. Lipid droplets were stained by incubating cells with BODIPY 493/503 (Invitrogen) for 30 min, followed by fixation and immunofluorescence processing as described above. Images of cells were acquired with a laser scanning confocal microscope (A1R VAAS with a 3 60/numerical aperture 1.49 oil-immersion lens; Nikon, Melville, NY, USA) and NIS-Elements AR software (Nikon). For all experiments, approximately 30 cells per sample were counted, and triplicate samples were counted for each experimental condition. Autophagy quantification was performed with the “Analyze Particle” function of ImageJ software (National Institutes of Health, Bethesda, MD, USA). To quantify co-localization, the ImageJ JACoP plug-in ref. 37 was used to calculate the percentage of co-localization from Manders’s overlapping coefficients (i.e., the fraction of red that overlaps green)^38^.

### Detection of acidic vesicular organelles: AVOs

To detect AVOs, MPH cells were cultured in a glass-bottomed dish in DMEM with 10% FBS and antibiotics. After treatment with lovastatin for 24 h, the cells were stained with 1 μM AO at 37 °C in the dark for 20 min. Next, the cells were washed with PBS and visualized using a laser-scanning confocal microscope (Fluoview FV1000; Olympus, Tokyo, Japan). Acridine orange stains DNA green in healthy cells and turns orange when it is sequestered into lysosomes and becomes protonated. To quantify the number of AVOs, the cells were harvested with trypsin treatment and washed with PBS. Next, the cells were analyzed by flow cytometry (FACSCalibur; Becton Dickinson, Franklin Lakes, NJ, USA), and the population frequencies were calculated using Cell Quest Pro software (Becton Dickinson) for Mac^®^ OS 9.

### Live Cell Imaging

PC-3 cells were cultured in Dulbecco’s modified Eagle’s medium (DMEM) and supplemented with 10% (v/v) fetal bovine serum (FBS) (GIBCO) and 1/100 (v/v) Pen/Strep (GIBCO). Cells were maintained at 37 °C with 5% CO_2_ for the duration of the experiment. For transfections, cells were grown to 80% confluency in four chambered cover-glass system (Lab-TekII, NUNC, USA) and co-transfected with 4 μg of LC3B-RFP plus pCMV- GFP and LC3B-RFP plus PNPLA8-GFP plasmid DNAs, respectively per chamber using lipofectamine 2000 reagent (Invitrogen) following the manufacturer’s instructions. After 24 h of transfection, cells were washed with a fresh complete DMEM. Cells were examined in an inverted microscope. Confocal images were obtained with a Zeiss LSM 780 laser confocal microscope (Carl Zeiss MicroImaging, Inc.) using a 63_oil-immersion objective equipped with an objective heater.

### Statistical analysis

Experiments were repeated at least three times with consistent results. Unless otherwise stated, data are expressed as the means ± standard deviations. An analysis of variance was used to compare experimental and control values. Comparisons between multiple groups were performed using Tukey’s multiple comparison test. Results were considered statistically significant at a p value of <0.05.

## Additional Information

**How to cite this article**: Kim, K.-Y. *et al.* SREBP-2/PNPLA8 axis improves non-alcoholic fatty liver disease through activation of autophagy. *Sci. Rep.* **6**, 35732; doi: 10.1038/srep35732 (2016).

## Supplementary Material

Supplementary Information

Supplementary Video S1a

Supplementary Video S1b
