# Corrigendum: SREBP-2/PNPLA8 axis improves non-alcoholic fatty liver disease through activation of autophagy

**DOI:** 10.1038/srep37794

**Published:** 2016-11-29

**Authors:** Kwang-Youn Kim, Hyun-Jun Jang, Yong Ryoul Yang, Kwang-Il Park, JeongKon Seo, Il-Woo Shin, Tae-Il Jeon, Soon-cheol Ahn, Pann-Ghill Suh, Timothy F. Osborne, Young-Kyo Seo

Scientific Reports 6: Article number: 3573210.1038/srep35732; published online: 10 21 2016; updated: 11 29 2016

The original version of this Article contained typographical errors in the spelling of the author Yong Ryoul Yang which was incorrectly given as Yong Ryul Yang.

These errors have now been corrected in the PDF and HTML versions of this Article.

