# Supplementary material for: SREBP-2/PNPLA8 axis improves non-alcoholic fatty liver disease through activation of autophagy
Source: Sci Rep. 2016 Oct 21;6:35732. doi: 10.1038/srep35732 (PMC5073315; doi:10.1038/srep35732)
Supplement: Supplementary Information [file srep35732-s1.pdf]

Manuscript Title:

SREBP-2/PNPLA8 axis improves non-alcoholic fatty liver disease through activation of autophagy

Author List:

Kwang-Youn Kim[1], Hyun-Jun Jang[1], Yong-Ryoul Yang[1], Kwang-Il Park[2], JeongKon Seo[3], Il-Woo Shin[4], Tae-Il Jeon[5], Soon-cheol Ahn[6], Pann-Ghill Suh[1*], Timothy F. Osborne[7*], and Young-Kyo Seo[1*]

Supplementary Table S1; Primers and Oligonucleotides used.

**qPCR primers for mRNA identification**		
	**Forward**	**Reverse**
**Pnpla1**	**ACCCAGATACCCCTCATTCC**	**CAGGCCCATATTCAGCACTC**
**Pnpla2**	**TGTGGCCTCATTCCTCCTAC**	**GATGTTGGTGGAGCTGTCCT**
**Pnpla3**	**GGGCTACGCTATGTCTGAGC**	**ATGTTGAAGAACGGGTGGAG**
**Pnpla4**	**TAACGCCCGGTTATGACTTC**	**TGCTGGCTAGGAGGACCTTA**
**Pnpla5**	**TGCTCCAATCTCCTGGACTT**	**GAGTGGCAAAATCGGTGACT**
**Pnpla6**	**TTATGCGGAAGGTGTCACAG**	**AGAGGGTAGGTGGGAATTGG**
**Pnpla7**	**CCTCCTTACCCTAGCCTTCG**	**GCAGAGGTATTCCCAACCAA**
**Pnpla8**	**TTCTGCTCCCAAGGGACTTA**	**GACTCGACGGCTTGCTTAAC**
**Srebp-2**	**TGGGAGAGTTCCCTGATTTG**	**GATAATGGGACCTGGCTGAA**
**Srebp-1c**	**CCGGGGAACTTTTCCTTAAC**	**GTTGTTGATGAGCTGGAGCA**
**Hmgcr**	**GCCAGCAATACCCAGAATGT**	**CAACAAGATCTGTGGCTGGA**
**Ldlr**	**TCCTGGAGATGTGATGGACA**	**GAGCCATCTAGGCAATCTCG**
**qPCR primers for manual ChIP**		
**Pnpla8**	ACCAAAATCCACCTCCGAT G	GATGGGGCCATTAACATG AC
**LDLr**	GAACTTCCCACTGCTGC	CACGCCCAGAG TCATTC
**siRNA Sequences**		
**PNPLA8**	1.GGUUGAAAUACAUAGAAAGAAAUGA<br>2.AACAUGCAACUAAGAACAACUUGTA<br>3.GUCCUAAACUGAUGUAGUUCAUCTG	

Supplementary Figure S1. Tracking TG in HepG2 cells.

a

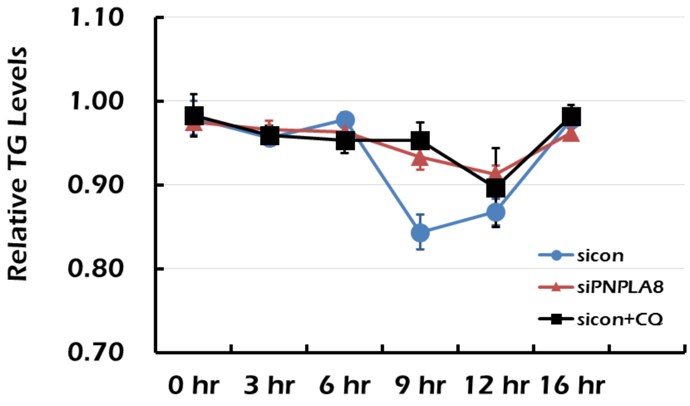

b

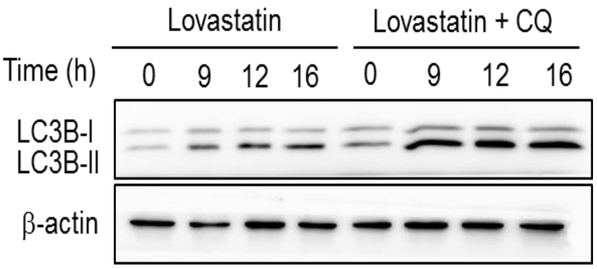

Supplementary Figure S1. After 24 hr of palmitic acid (400 uM, Sigma-Aldrich) treatment, HepG2 cells were treated with lovastatin (5 uM) or chloroquine (50 uM) treatment. Endogenous triglyceride levels from si PNPLA8, si control, and sicontrol + chloroquine treated HepG2 cells were measured over the time points of 0 to 16 hr (**a**). Consistent with TG clearance by autophagy formation, LC3IIB levels were increased over the time points in chloroquine treatment (**b**).

Supplementary Figure S2. SREBP-2 upregulates PNPLA8 expression in mouse liver.

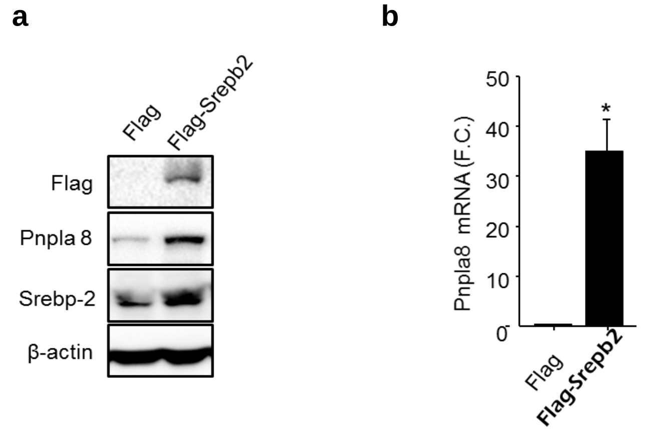

Supplementary Figure S2. A control vector (Flag) or a Flag-SREBP-2 expression vector were transfected into HEK293 cells and SREBP-2 and PNPLA8 protein (**a**) and PNPLA8 RNA (**b**) levels were measured (F.C.; fold change).All data are representative of three independent experiments. Data are shown as means ± standard deviations. *p < 0.01.

Supplementary Figure S3. Tracking of the functional properties of Pnpla8 *in vivo.*

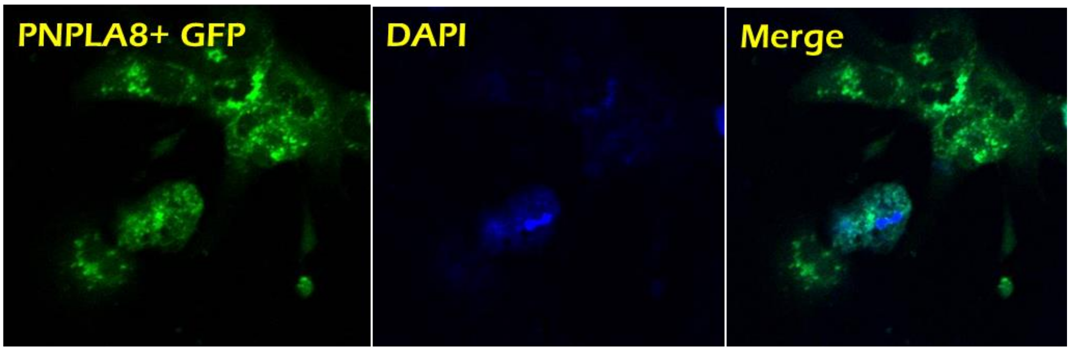

Supplementary Figure S3. The GFP-PNPLA8 expressing plasmid were injected into the tail veins of 12 weeks-HFD mice. Mice were sacrificed and GFP expression was analyzed from the primary hepatocytes.

Supplementary Figure S4. Full unedited blots.

Figure 2.

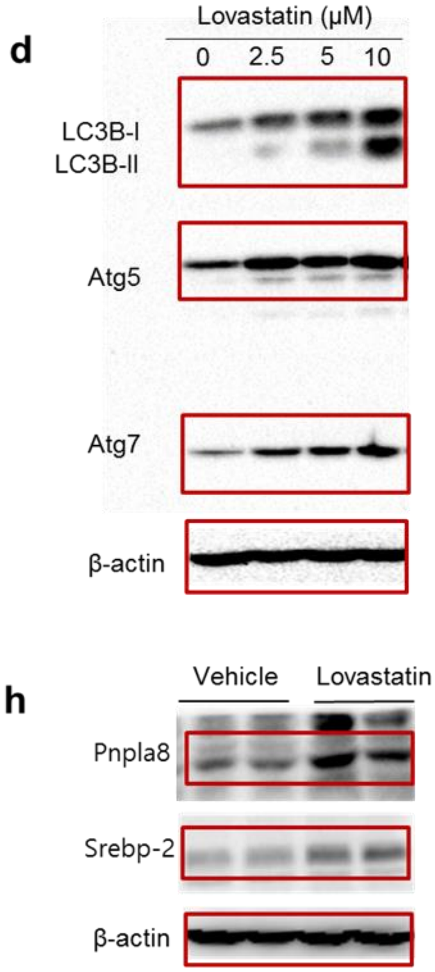

Supplementary Figure S4. (**Figure 2d h**, **Figure 3b d e f**, **Figure 5f**, **Figure 7a**, **Supplementary Figure S1**) Red boxes indicate images shown in the corresponding Figures.

Supplementary Figure S4 continued.

Figure 3.

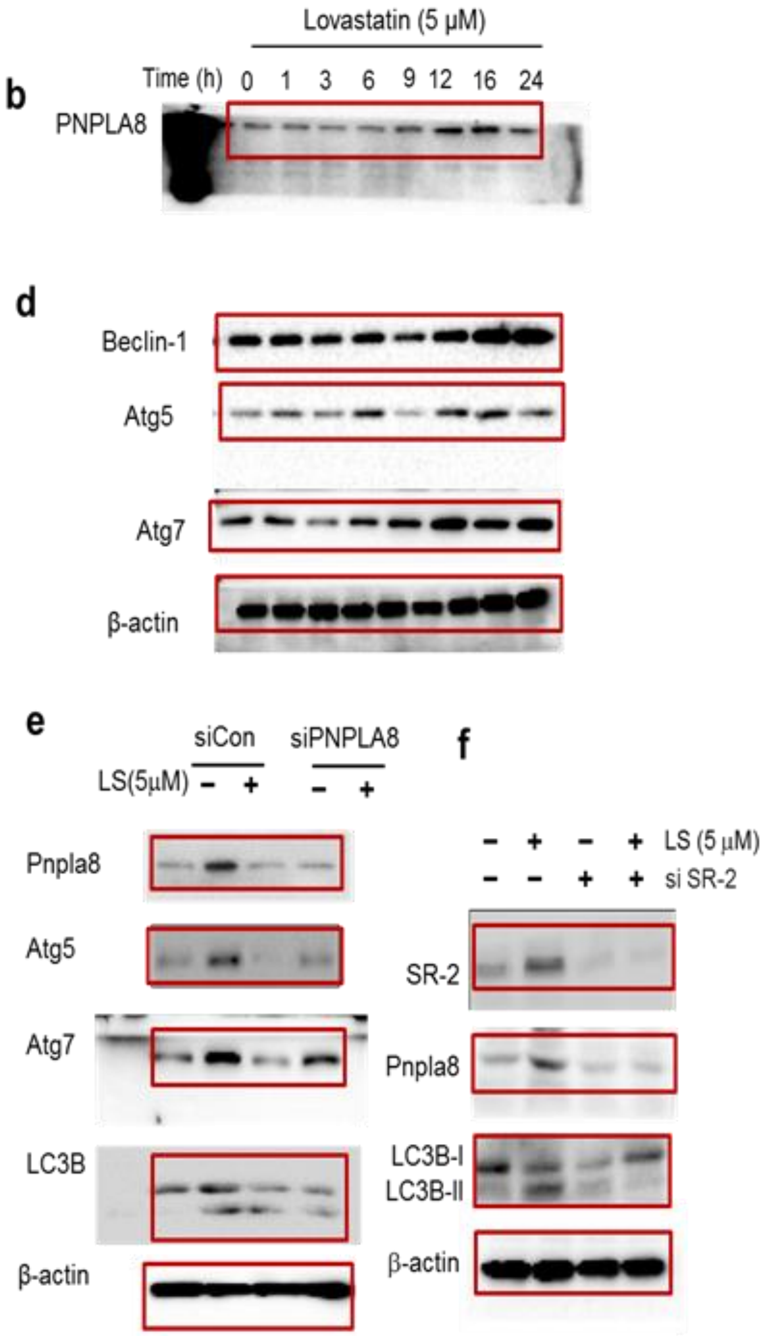

Supplementary Figure S4 continued.

Figure 5f.

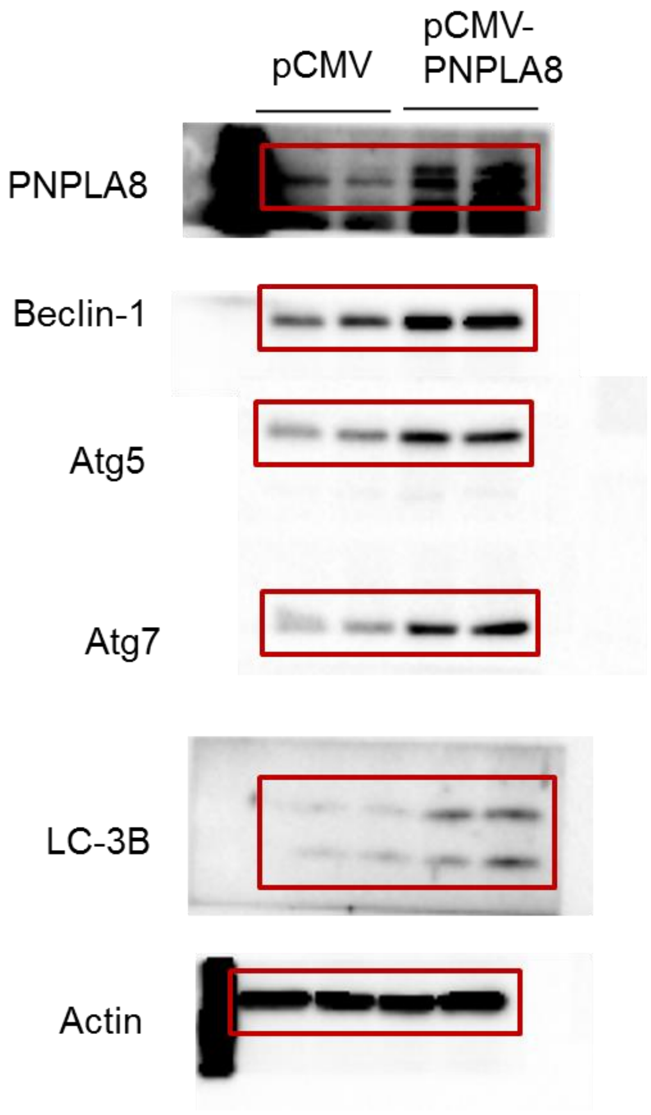

Supplementary Figure S4 continued.

Figure 7a.

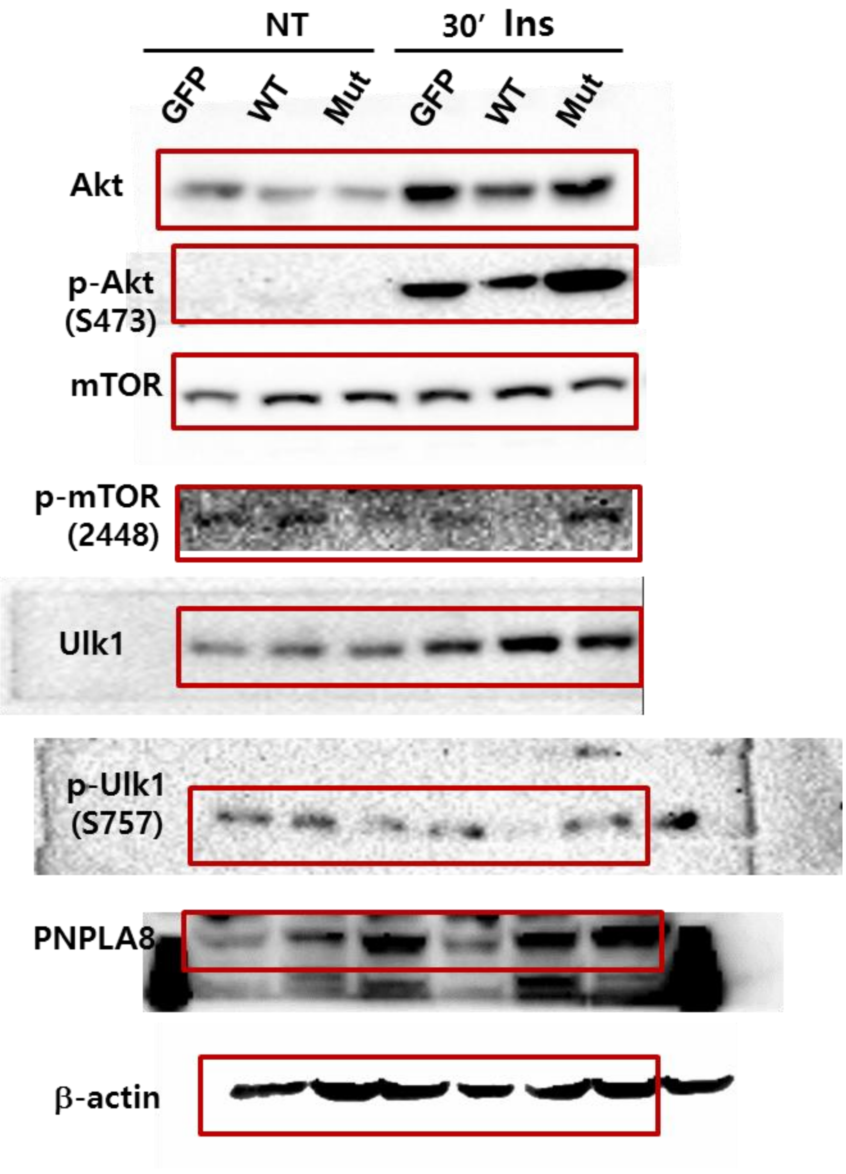

Supplementary Figure S4 continued.

Supplementary Figure S1.

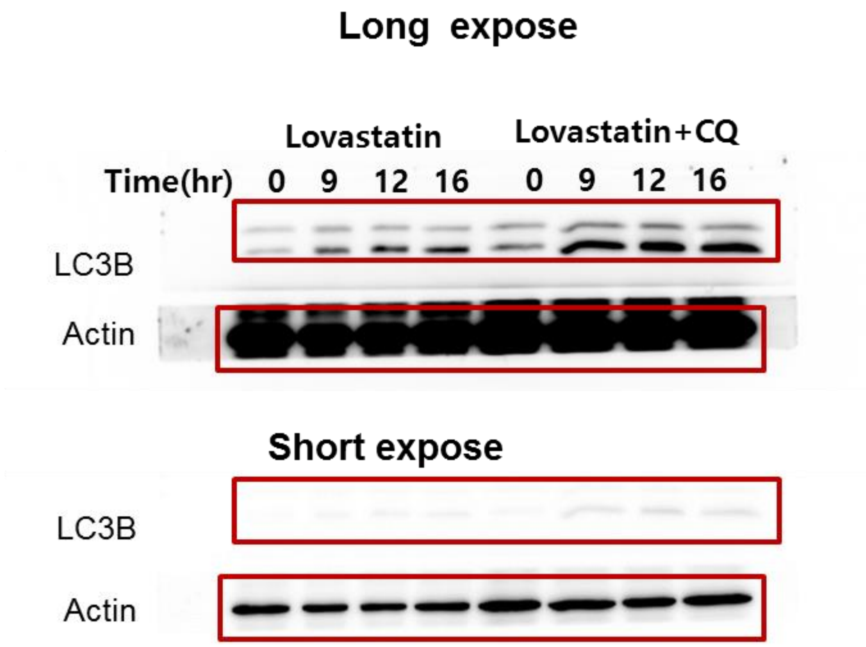

Supplementary Video S1. Dynamic interactions between PNPLA8 and LC3.

a Uploaded as separate video file.

b Uploaded as separate video file.

Supplementary Video S1. (**a**) PC-3 cells were transfected were with pCMV**+**PNPLA8-GFP (green) or pCMV+LC3B (red) and imaged by confocal microscopy (LSM780NLO' Carl Zeiss, Germany) in time lapse. (**b**) pCMV+GFP or pCMV+LC3B as a control movies were imaged identically. Images at 3 frames per second are shown. Scale bar, 10 μm.
